# Role of Intraoperative Bronchoscopy in Diagnosing Bronchus Related Complications Following VATS

**DOI:** 10.4274/TJAR.2026.252260

**Published:** 2026-04-15

**Authors:** Sathish K, Senthilkumar Sukumar, Raja Siddhartha K S

**Affiliations:** 1 MGM Cancer Institute, Consultant, Department of Anaesthesiology & SICU, Chennai, India; 2 MGM Cancer Institute, Department of Anaesthesiology & SICU, Chennai, India

**Keywords:** Diagnosis, lung neoplasms, adverse effects, bronchoscopy, video-assisted thoracic surgery

## Abstract

We report the case of a 60-year-old female with adenocarcinoma of the right upper lobe who underwent a video-assisted thoracoscopic surgery (VATS) upper lobectomy and subsequently presented with complete right lung collapse in the immediate postoperative period. Urgent bronchoscopy revealed complete stapling of the right mainstem bronchus. Hence, emergency re-exploration and bronchoplasty of the right mainstem bronchus and the right lower-lobe bronchus were done. While bronchoscopy following VATS lobectomy for lung cancer poses technical challenges and represents an independent risk factor for postoperative pulmonary complications, it remains a valuable tool for the early detection of complications such as lung collapse, which may result from thick mucus, a foreign body, iatrogenic injury, or the tumour itself.

**Main Points**

• In this case, despite the risk of postoperative pulmonary complications, bronchoscopy after video-assisted thoracoscopic surgery facilitated early detection of surgical complication.

• In this case, postoperative bronchoscopy revealed a completely stapled right mainstem bronchus.

• Re-exploration and bronchoplasty of the right mainstem bronchus and of the lower lobe bronchus were performed as corrective surgery.

• Perioperative bronchoscopy is a valuable tool for the early detection of complications, in this case detecting a lung collapse related to a surgical staple.

## Introduction

Evidence-based studies show that airway complications after lobectomy are associated with significant morbidity and mortality.^1^ Pulmonary lobar torsion, bronchovascular fistula, bronchopleural fistula, and atelectasis^2^ present a significant clinical challenge, warranting early diagnosis and prompt intervention to improve patient outcomes.

Bronchoscopy facilitates earlier detection of such complications^3^ compared with imaging, which is time-consuming and requires careful review of reports. The present case report emphasizes the importance of intraoperative bronchoscopy following any lung isolation procedure, enabling the early recognition and effective management of complications.

## Case Report

**Figure 1:**
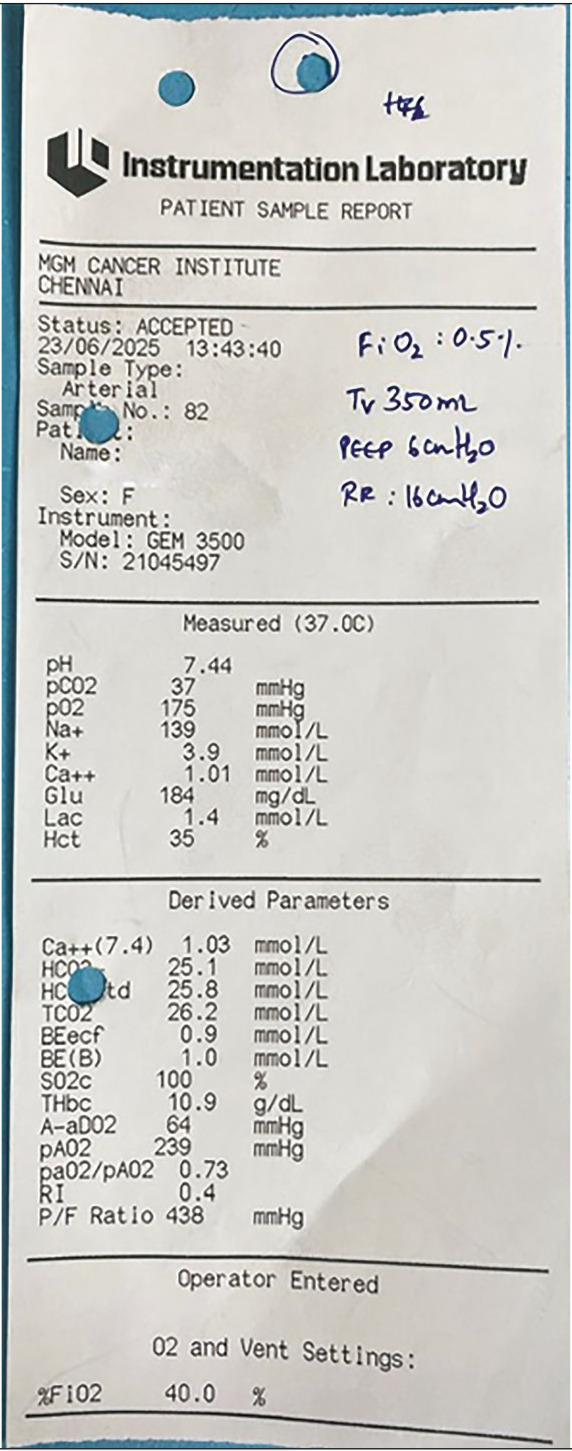
ABG prior to extubation. ABG, arterial blood gas.

The informed consent was duly acquired from the patient. A 60-year-old female patient with a known history of asthma and hypertension was diagnosed with adenocarcinoma of the right lung and was scheduled for a video-assisted thoracoscopic surgery (VATS) right upper lobectomy. Computed tomography (CT) revealed a nodule of 3×2 cm in the posterior segment of the right upper lobe. After a comprehensive assessment and optimisation with steroids and bronchodilator coverage for asthma, a VATS-assisted right upper lobectomy was planned. After routine induction of anaesthesia, lung separation was achieved using a 35-F left-sided double-lumen tube (DLT) guided by fibreoptic bronchoscopy, and surgery proceeded. A uniportal VATS right upper lobectomy was successfully performed, and a functional intercostal drain was placed. The DLT was subsequently replaced with a single-lumen tube,^4^ and adequate lung recruitment was achieved. Arterial blood gas analysis showed a satisfactory PaCO_2_ of 37 mmHg and a P/F ratio of 430 (Figure 1).

**Figure 2:**
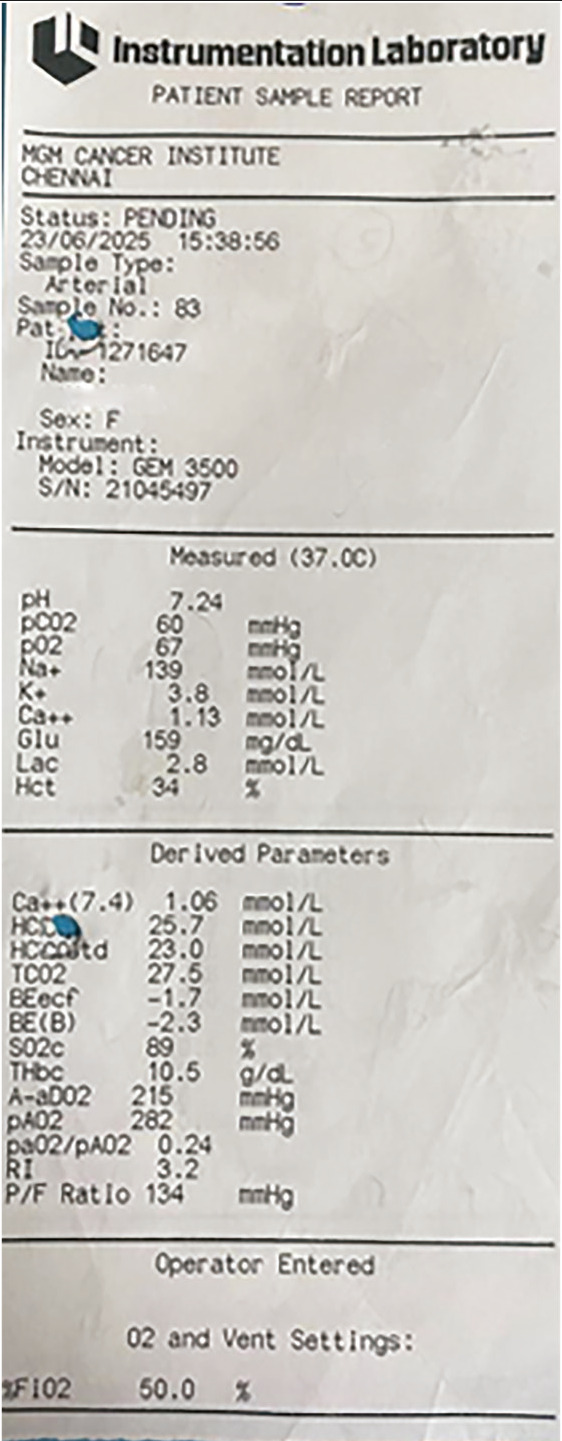
Postoperative ABG in ICU. ABG, arterial blood gas; ICU, intensive care unit.

**Figure 3:**
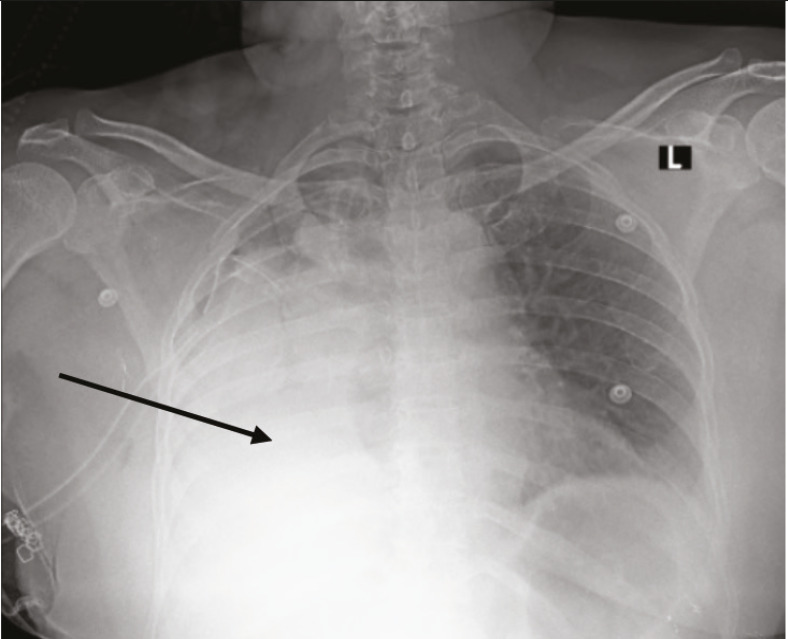
Postoperative chest radiograph showing total collapse of the right hemithorax. L: left.

Following adequate analgesia, neuromuscular blockade was reversed, and the trachea was extubated. Post-extubation, the patient complained of pain and difficulty in breathing ; during this episode, oxygen saturation dropped from 100% to 92% despite administration of 100% oxygen. After additional analgesic supplementation, auscultation revealed decreased, coarse breath sounds with rhonchi over the right hemithorax. Bronchospasm was suspected, and treatment with bronchodilators and steroids was initiated. Oxygen saturation subsequently improved to 96-98% with an FiO_2_ of 1.0. Given the diagnosis of bronchospasm and established hypoxic pulmonary vasoconstriction (HPV), the patient was transferred to the post-surgical intensive care unit (ICU) for oxygen therapy via high-flow nasal oxygen (HFNO) and bronchodilator therapy. After 30 minutes of HFNO with an FiO_2_ of 0.8, the arterial PCO_2 _was 60 mmHg, the arterial PO_2_ was 67 mmHg, and the P/F ratio was 134 (Figure 2). A postoperative chest radiograph displayed total collapse of the right hemithorax (Figure 3).

**Figure 4:**
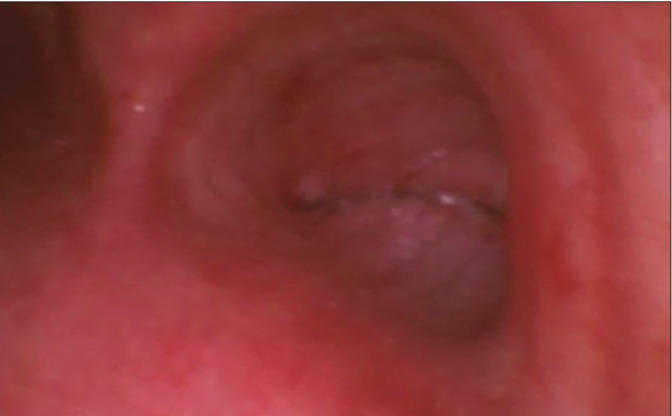
Bronchoscopy view demonstrating complete stapling of the right main bronchus.

Furthermore, bronchoscopy was performed immediately, revealing a complete obstruction of the right main bronchus (Figure 4). A diagnosis of accidental stapling of the right main bronchus was established (Supplementary Video 1), and the patient was immediately taken back to the operating room for re-exploration. Complete occlusion of the right main bronchus with collapse of the middle and lower lobes was noted. Bronchoplasty of the right main bronchus and the lower lobe bronchus was performed, along with middle lobectomy, because of a narrowed bronchial opening. Bronchial patency was confirmed using a paediatric bronchoscope (Ambu Slim, 3.8 mm) before extubation.

**Figure 5:**
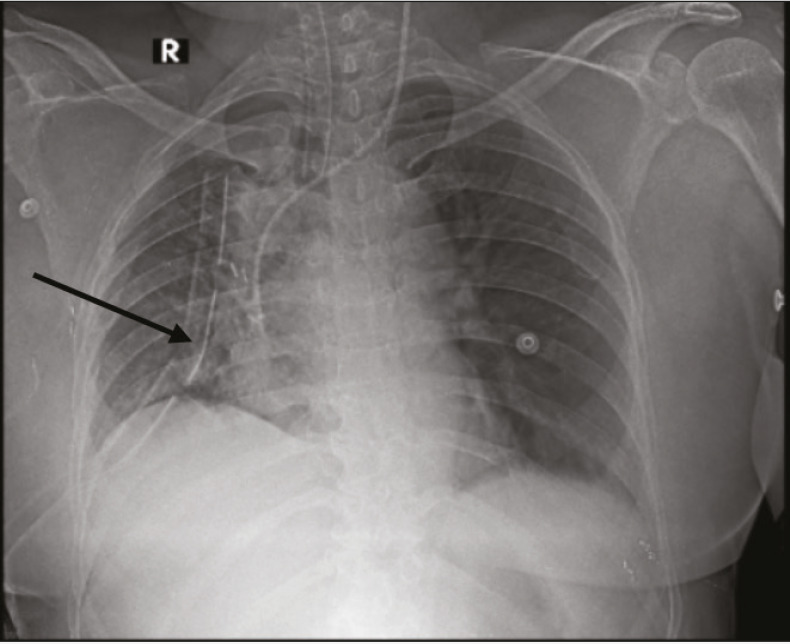
Postoperative chest X-ray after re-exploration, showing re-inflation of the right lower lobe. R: right.

The patient was transferred to the postoperative ICU for elective ventilation via a single-lumen tube and was extubated 12 hours later after a postoperative X-ray (Figure 5) confirmed expansion of the right lower lobe.

## Discussion

VATS is a minimally invasive surgical technique that offers several advantages over traditional open surgery. However, as with any  surgical procedure, it is associated with potential risks and complications, including bleeding, infection, pneumonia, pneumothorax, and prolonged air leak. Among these, bronchial injuries account for approximately 0.1-1.5% of cases^5, 6^ and are typically caused by surgical intervention or stapler closure of the bronchial stump.^7^ Early diagnosis, guided by a high index of clinical suspicion, is crucial for reducing morbidity and mortality.^2, 7^ Bedside lung point-of-care ultrasound can be used, but only as an adjunct to radiography for diagnosing early respiratory complications.^8^ In such clinical scenarios, CT imaging and bronchoscopy play a pivotal role in confirming the diagnosis.

Intraoperative diagnostic bronchoscopy is not routinely performed unless there is a clear indication, because it is associated with an increased risk of postoperative pulmonary complications and prolonged hospital stay among patients undergoing lobe resection for lung cancer.^9^

In the current case, no significant hypoxia or abnormally elevated airway pressures were observed intraoperatively after initiation of dual-lung ventilation that would suggest complete bronchial obstruction.

Following extubation patient was shifted to ICU where the patient developed marked hypoxia requiring HFNO. A chest radiograph revealed total collapse of the right hemithorax; bronchoscopy was immediately undertaken to evaluate a presumptive diagnosis of mucus plugging or inadequate recruitment. However, bronchoscopy revealed that the right main bronchus had been inadvertently stapled. Although perioperative bronchoscopy remains technically challenging due to blood and secretions in the airway, which compromise visibility and increase infection risk, it may be used as a valuable bedside tool for detecting early complications.^10, 11^ Based on the clinical presentation, poor recruitment, mucus plugging, and HPV were considered  differential diagnoses until bronchoscopy was performed. Intraoperative bronchoscopy plays a crucial role in identifying an off-target bronchial transection in the remnant neighbouring lobes, including the visualisation of ipsilateral adjacent airway and the localisation of surgical ligation clips. In such cases, bronchoscopy serves as an essential tool to prevent diagnostic delay. Hence, we began performing post-procedure bronchoscopy and lung ultrasound to assess bronchial patency and lung expansion, respectively, prior to extubation in all such cases.

## Conclusion

To sum up, although complications following the VATS procedure are rare, they can be fatal. In such instances, intraoperative bronchoscopy, despite its technical difficulty, serves as a valuable bedside tool for the diagnosis of immediate postoperative complications.

## Ethics

**Informed Consent:** The informed consent was duly acquired from the patient.

## Supplementary Materials

Supplementary Video 1 https://youtu.be/H4NOP3i_F-Y
